# Practical synthesis of 1,3-benzoazaphosphole analogues

**DOI:** 10.3389/fchem.2023.1174895

**Published:** 2023-05-25

**Authors:** Yuki Yamamoto, Soichiro Mita, Yuki Sato, Kentaro Yano, Akiya Ogawa

**Affiliations:** ^1^ Department of Applied Chemistry, Graduate School of Engineering, Osaka Prefecture University, Sakai, Osaka, Japan; ^2^ Functional Dye Division, Hayashibara Co., Ltd., Minami-ku, Okayama, Japan

**Keywords:** 1,3-benzoazaphosphole analogues, 2-aminophenyl(phenyl)phosphine, practical synthesis route, stable phosphorus key intermediate, heterocycles

## Abstract

Despite the promising pharmacological activity and material properties of five-membered heterocyclic compounds containing phosphorus and nitrogen, synthetic examples of them have been rather limited due to the instability of phosphorus toward air and water. In this study, 1,3-benzoazaphosphol analogues were selected as target molecules, and various synthetic routes were examined to establish a fundamental technology for the introduction of phosphorus groups into aromatic rings and formation of five-membered rings containing phosphorus and nitrogen by cyclization. As a result, we found that 2-aminophenyl(phenyl)phosphine is an extremely promising synthetic intermediate with high stability and easy handling. Furthermore, 2-methyl-3-phenyl-2,3-dihydro-1*H*-benzo[*d*][1,3]azaphosphole and 3-phenyl-2,3-dihydro-1*H*-benzo[*d*][1,3]azaphosphole-2-thione as synthetically useful 1,3-benzoazaphosphol analogues were successfully synthesized by using 2-aminophenyl(phenyl)phosphine as a key intermediate.

## 1 Introduction

**SCHEME 1:**
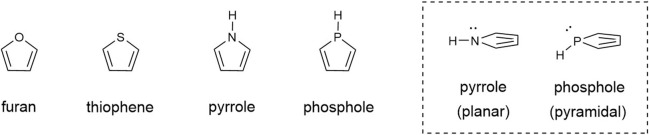
Five-membered heterocycles and their structural features.

Furan, pyrrole, thiophene, and their saturated compounds are representative five-membered heterocyclic compounds with good stability, and numerous derivatives have been synthesized. These heterocyclic compounds are indispensable in medical and agrochemical as well as materials chemistry (Baumann, et al., 2011; Jangir, et al., 2022). In sharp contrast, five-membered ring heterocyclic compounds containing phosphorus are more unstable than *O*-, *N*-, and *S*-heterocycles. For example, the three bonds around the nitrogen atom of pyrrole are planar, whereas the bonds around the phosphorus atom of phosphole, in which the nitrogen atom of pyrrole is replaced by a phosphorus atom, are pyramidal (Scheme 1). As a result, phosphole exhibits very low aromaticity (Egan, et al., 1971; Epitotis, et al., 1976; Mathey, et al., 1988; Cyrañski, et al., 2002; Chesnut, et al., 2007). Because of such a structural feature, conjugated molecules involving phosphole and its analogs have recently attracted attention in the field of organic semiconductor development, including light-emitting and electronic materials (Hay, et al., 2001; Hissler, et al., 2003; Baumgartner, et al., 2004; Su, et al., 2006; Hobbs, et al., 2007; Crassous, et al., 2008; Matano, et al., 2009; Ren, et al., 2012). Despite their promising material properties, synthetic examples of five-membered ring heterocyclic compounds containing phosphorus are rather limited.

**SCHEME 2:**
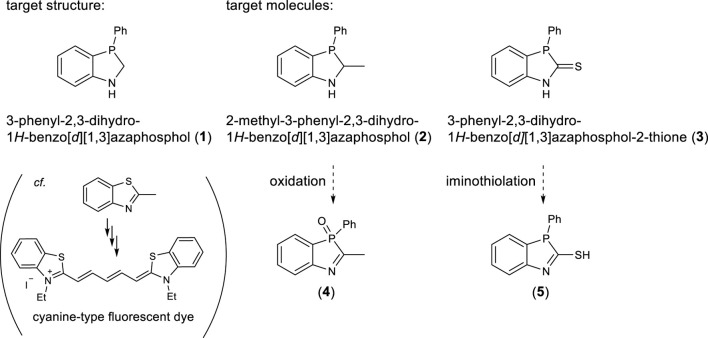
1,3-Benzoazaphosphole analogs synthesized in this study.

In order to take advantage of the properties of phosphorus and to realize diversity in combination with other heteroatoms, it is extremely desired to establish a fundamental technology for the formation of five-membered rings containing phosphorus. From this viewpoint, in this study, we have studied the development of synthetic methods for the formation of five-membered heterocyclic rings containing phosphorus and nitrogen. In particular, we selected 3-phenyl-2,3-dihydro-1*H*-benzo[*d*][1,3]azaphosphole **1** as a target structure and specifically investigated the synthesis of 2-methyl-3-phenyl-2,3-dihydro-1*H*-benzo[*d*][1,3]azaphosphole **2** and 3-phenyl-2,3-dihydro-1*H*-benzo[*d*][1,3]azaphosphole-2-thione **3** in detail, that have never been synthesized before. Since 2-methylbenzothiazole, in which the PhP group of **2** is replaced with S, can be derived into a cyanine-type fluorescent dye using the methyl group at the 2-position as a clue, **2** might be a key intermediate for cyanine-type luminescent dyes bearing a 1,3-azaphosphole unit. In addition, **3** is expected to lead to an azaphosphole having a thiol group at the 2-position by iminothiolation and might cause bond-connection through sulfur (Scheme 2).

## 2 Materials and methods

### 2.1 Synthesis of ethyl (2-aminophenyl)(phenyl)phosphinate via Pd-catalyzed cross-coupling reaction

2-Bromoaniline **6a** (18 mmol, 3096.5 mg), ethyl phenylphosphinate **23** (17 mmol, 2892.6 mg), Pd(OAc)_2_ (2 mol%, 76.3 mg), dppf (2.2 mol%, 207.3 mg), ^*i*^Pr_2_NEt (22.4 mmol, 3.8 mL), degassed DMF (72 mL), and dehydrated ethylene glycol dimethyl ether (8 mL) were added to a 300-mL three-neck round-bottom flask under an argon atmosphere, and the solution was heated at 115°C for 24 h. After the reaction, the solvents were removed under reduced pressure. AcOMe (50 mL) was added to the residue, and the organic layer was washed with saturated NaHCO_3_ aq. (30 mL×2). The water layer was extracted with AcOMe (40 mL×3), and the combined organic layer was washed with brine (30 mL), dried with anhydrous Na_2_SO_4_, and filtered. The filtrate was concentrated under reduced pressure, and the residue was purified by silica-gel column chromatography (eluent: *iso*-hexane/AcOMe = 7 : 3 with 5% Et_3_N) to obtain pure ethyl (2-aminophenyl)(phenyl)phosphinate **24** as brown oil in 62% isolated yield (Eq. 11).

*Ethyl (2-aminophenyl)(phenyl)phosphinate* (**24**) [CAS no. 93383–23-4] (Kyba, et al., 1984). Brown oil, 2741.9 mg, 62%; ^1^H NMR (400 MHz, CDCl_3_): δ 7.85–7.75 (m, 2H), 7.43–7.29 (m, 4H), 7.14 (t, *J* = 8.0 Hz, 1H), 6.55–6.64 (m, 2H), 5.57 (bs, 2H), 4.18–4.00 (m, 2H), 1.31 (t, *J* = 7.0 Hz, 3H); ^13^C{^1^H} NMR (100 MHz, CDCl_3_): δ 161.9, 151.5 (d, $J_{C–P}=7.0$ Hz), 133.0, 132.2 (d, $J_{C–P}=9.0$ Hz), 131.9 (d, $J_{C–P}=139.0$ Hz), 131.4, 130.5 (d, $J_{C–P}=10$ Hz), 127.8 (d, $J_{C–P}=13.0$ Hz), 116.0 (d, $J_{C–P}=10$ Hz), 110.0 (d, $J_{C–P}=138.0$ Hz), 60.5 (d, $J_{C–P}=6$ Hz), 15.9 (d, $J_{C–P}=7$ Hz); ^31^P NMR (162 MHz, CDCl_3_): δ 36.1.

### 2.2 Reduction of ethyl (2-aminophenyl)(phenyl)phosphinate with DIBAL-H

Ethyl (2-aminophenyl)(phenyl)phosphinate **24** (14 mmol) dissolved in dehydrated Et_2_O (15 mL) was transferred to a 500-mL three-neck flask, and DIBAL-H (1.0 M in hexane, 7.0 equiv.) was slowly added to the reaction mixture at 0°C for 2 h, and the reaction mixture was stirred at 25°C for 70 h. After the reaction, K_2_HPO_4_ aq. (0.5 M, 140 mL) was slowly added to the reaction vessel at 0°C for 1 h. The mixture was extracted with Et_2_O (50 mL×5), and the combined organic layer was washed with brine (40 mL), dried with anhydrous MgSO_4_, and filtered. The filtrate was concentrated under reduced pressure, and the residue was purified by silica-gel column chromatography (eluent: *iso*-hexane/AcOMe = 9 : 1 with 5% Et_3_N) to obtain 2-(phenylphosphanyl)aniline (**25)** in 91% ^31^P NMR yield. Since the highly concentrated **25** could be potentially oxidized with air, the obtained **25** was stored under an argon atmosphere and directly used for the following reactions without any further purification (Table 2).

*2-(Phenylphosphanyl)aniline* (**25**) [CAS no. 67405–21-4] (Bennett et al., 2006). Pale yellow oil. ^1^H NMR (400 MHz, CDCl_3_): δ 7.45–7.36 (m, 3H), 7.30–7.22 (m, 3H), 7.21–7.16 (m, 1H), 6.77–6.70 (m, 1H), 6.68–6.61 (m, 1H), 5.11 (d, *J*_P–H_ = 222 Hz, 1H), 3.89 (bs, 2H); ^13^C{^1^H} NMR (100 MHz, CDCl_3_): δ 149.5 (d, $J_{C–P}=8$ Hz), 137.6 (d, $J_{C–P}=22$ Hz), 133.5 (d, $J_{C–P}=10$ Hz), 132.4 (d, $J_{C–P}=15$ Hz), 131.2, 118.5 (d, $J_{C–P}=8$ Hz), 115.9 (d, $J_{C–P}=10$ Hz), 115.4; ^31^P NMR (162 MHz, CDCl_3_): δ −59.4.

### 2.3 Synthesis of 2-methyl-3-phenyl-2,3-dihydro-1*H*-benzo[*d*][1,3]azaphosphole

2-(Phenylphosphanyl)aniline **25** (1.0 mmol), acetaldehyde (1.5 mmol, 90 wt% aq.), 4A MS (100 mg), and degassed toluene (1 mL) were placed in a 10-mL Schlenk tube, and the mixture was refluxed at 120°C for 48 h. After the reaction, the resulting solution was filtered, and the filtrate was concentrated under reduced pressure. Finally, the residue was purified by gel permeation chromatography (eluent: CH_2_Cl_2_) to obtain 2-methyl-3-phenyl-2,3-dihydro-1*H*-benzo[*d*][1,3]azaphosphole (**2)** in 82% ^31^P NMR yield (*anti*/*syn* = 49/51) (Eq. 14).

2-Methyl-3-phenyl-2,3-dihydro-1H-benzo[d][1,3]azaphosphole (**2**).

(*Anti*-isomer) Light yellow oil; ^1^H NMR (400 MHz, CDCl_3_): δ 7.46–7.42 (m, 1H), 7.34–7.20 (m, 4H), 7.16–7.10 (m, 2H), 6.83–6.75 (m, 2H), 4.40–3.90 (m, 2H), 1.14–1.07 (m, 3H); ^13^C{^1^H} NMR (100 MHz, CDCl_3_) δ 155.7, 133.7 (d, $J_{C–P}=19$ Hz), 133.6 (d, $J_{C–P}=27$ Hz), 132.3 (d, $J_{C–P}=22$ Hz), 130.8, 129.2, 128.1 (d, $J_{C–P}=6.7$ Hz), 123.6 (d, $J_{C–P}=5.8$ Hz), 119.0 (d, $J_{C–P}=7.7$ Hz), 110.0, 54.7 (d, $J_{C–P}=12$ Hz), 15.7; ^31^P NMR (162 MHz, CDCl_3_): δ −18.2; HRMS (EI) *m/z* calcd for C_14_H_14_NP [M]^+^:227.0864, found: 227.0870.

(*Syn*-isomer) Pale yellow oil. ^1^H NMR (400 MHz, CDCl_3_): δ 7.52–7.46 (m, 1H), 7.30–7.20 (m, 6H), 6.83–6.74 (m, 2H), 4.07 (bs, 1H), 3.90–3.83 (m, 1H), 1.42–1.34 (m, 3H); ^13^C{^1^H} NMR (100 MHz, CDCl_3_): δ 153.9, 139.0 (d, $J_{C–P}=22$ Hz), 132.6 (d, $J_{C–P}=22$ Hz), 131.5 (d, $J_{C–P}=22$ Hz), 130.9, 128.4 (d, $J_{C–P}=6.7$ Hz), 120.7 (d, $J_{C–P}=8.7$ Hz), 118.6 (d, $J_{C–P}=7.7$ Hz), 110.1, 57.5 (d, $J_{C–P}=12$ Hz), 22.2 (d, $J_{C–P}=31$ Hz); ^31^P NMR (162 MHz, CDCl_3_): δ −6.9; HRMS (EI) *m/z* calcd for C_14_H_14_NP [M]^+^:227.0864, found: 227.0870.

### 2.4 Synthesis of 3-phenyl-1,3-dihydro-2*H*-benzo[*d*][1,3]azaphosphole-2-thione

2-(Phenylphosphanyl)aniline **25** (1.5 mmol), 1,1′-thiocarbonyldiimidazole (1.5 mmol), and degassed THF (5 mL) were placed in a 10-mL Schlenk tube, and the mixture was heated at 80°C for 48 h. After the reaction was completed, the solvent was removed under reduced pressure, and the residue was purified by gel permeation chromatography (eluent: CH_2_Cl_2_) to obtain 3-phenyl-1,3-dihydro-2*H*-benzo[*d*][1,3]azaphosphole-2-thione (**3)** in 97% ^31^P NMR yield (Eq. 18).

*3-Phenyl-1,3-dihydro-2H-benzo[d][1,3]azaphosphole-2-thione* (**3**). Yellow solid; ^1^H NMR (400 MHz, CDCl_3_): δ 11.2 (bs, 1H), 7.50–7.44 (m, 3H), 7.40–7.30 (m, 4H), 7.24–7.18 (m, 1H), 7.13 (d, *J* = 8 Hz, 1H); ^13^C{^1^H} NMR (100 MHz, CDCl_3_): δ 213.7 (d, $J_{C–P}=31$ Hz), 146.4 (d, $J_{C–P}=3$ Hz), 132.7 (d, $J_{C–P}=19$ Hz), 130.6 (d, $J_{C–P}=17$ Hz), 130.3, 129.1 (d, $J_{C–P}=7$ Hz), 127.9 (d, $J_{C–P}=11$ Hz), 124.8 (d, $J_{C–P}=6$ Hz), 112.4; ^31^P NMR (162 MHz, CDCl_3_): δ 15.8; HRMS (EI) *m/z* calcd for C_13_H_10_NPS [M]^+^:243.0272, found: 243.0276.

## 3 Results and discussion

**SCHEME 3:**
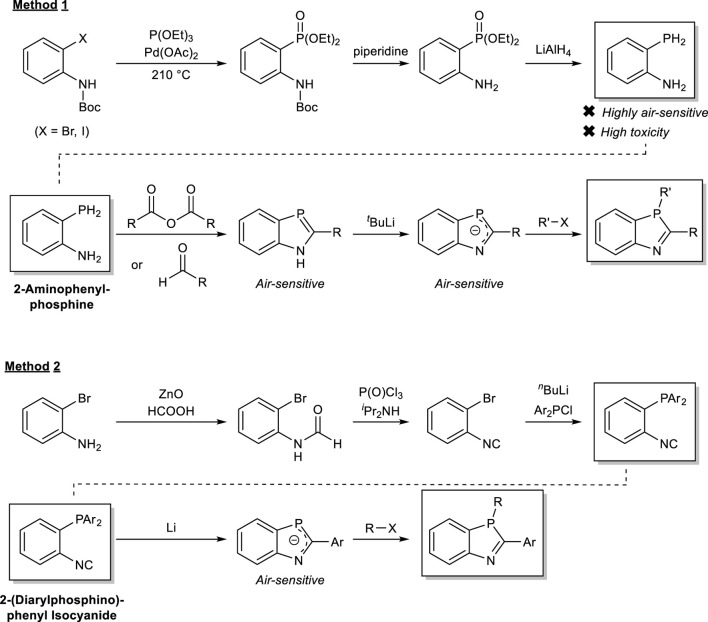
Conventional methods for the synthesis of benzoazaphospholes.

Five-membered ring heterocyclic compounds with phosphorus and nitrogen have been largely limited in their synthetic routes compared to other five-membered ring heterocyclic compounds incorporating nitrogen, oxygen, or sulfur due to the instability of the trivalent phosphorus functional groups with air and water, the toxicity of the phosphorus–hydrogen compounds as synthetic intermediates, and the difficulty in handling the key intermediates for such phosphorus–nitrogen heterocycles. Therefore, the use of five-membered ring heterocyclic compounds with phosphorus and nitrogen in materials chemistry has been significantly restricted. Thus, the development of versatile synthetic methods is strongly desired. As to 1,3-benzophospholes, for example, only two methods have mainly been used for their synthesis (Scheme 3).

Method 1 mainly uses a route with the key intermediate 2-aminophenylphosphine, which is highly oxidizable and highly toxic. Although this route gives high yields for each step, except the final alkylation (∼60%), it requires hazardous purification of 2-aminophenylphosphine by distillation and use of a pyrophoric base (^*t*^BuLi). Unfortunately, the substituent on the phosphorus atom is limited to an alkyl group (Issleib, et al., 1978; Walborsky, et al., 1978; Issleib, et al., 1981; Heinicke, et al., 1982; Heinicke, 1986; Rösch, et al., 1987; Heinicke, 1989; Bansal, et al., 1999; Niaz, et al., 2013; Niaz, et al., 2013). Method 2 involves the synthesis of *P*-substituted 2-aryl-1,3-benzazaphospholes using 2-(diarylphosphino)phenyl isocyanide as a key intermediate. In this route, the key intermediate isocyanide is air-stable, but suffers from low yields of phosphorus functionalization and cyclization (both 60%) and restriction of the 2-position of the 1,3-azaphosphole ring to an aryl group (Zhang, et al., 2015).

Since Method 1 requires toxic and air-sensitive 2-aminophenylphosphine as a key intermediate, we synthesized 1,3-azaphosphole derivatives by modifying Method 2. Treatment of 2-bromoaniline **6a** with ZnO/HCOOH afforded the formation of the corresponding formamide **7** in 92% yield, which led to formation of 2-bromophenyl isocyanide **8** in 82% yield by following dehydration (Eq. 1). However, the Br–Li exchange reaction of **8** and the following phosphination with Ph_2_PCl required strict temperature control for inhibiting isocyanide oligomerization.

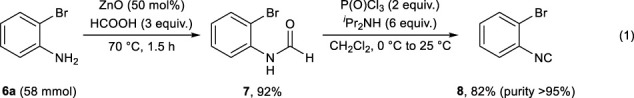

(1)



Thus, we next selected the imino group instead of the isocyano group because the imino group is not susceptible to oligomerization. Before the imination reaction, the synthesis of 2-aminophosphinic ester was examined by rearrangement of the phosphino group. Then, LiAlH_4_ reduction followed by imination might lead to the formation of 1,3-azaphosphole. The reaction of 2-iodoaniline **9** with phenylphosphonic dichloride **10** successfully afforded 2-iodophenylaminophosphinic ester **11** as the major product. However, the following lithiation from **11** did not proceed well (Eq. 2).

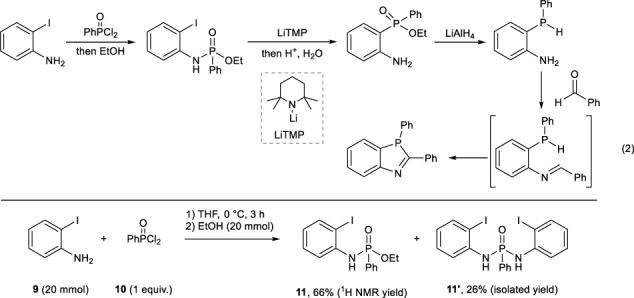

(2)



As described previously, the synthesis of arylphosphorus compounds via aryllithium intermediates requires a high level of synthesis techniques and is considered undesirable from the standpoint of versatility. Therefore, we next examined the synthesis of arylphosphorus compounds by transition-metal-catalyzed coupling reactions. A proposed synthesis pathway is shown in Eq. 3. Although PdCl_2_-catalyzed coupling of 2-iodoaniline **9** with phenylphosphonic dichloride **10** afforded 5% of the desired 2-aminophenyl(phenyl)phosphine oxide **12** along with its dimer **12'** (35%) due to the nucleophilic attack of the amino group at the phosphine, copper(I) iodide-catalyzed coupling reaction of 2-iodoaniline **9** with diphenylphosphine **14** successfully afforded 2-aminophenyl(diphenyl)phosphine **15** in 42% yield.

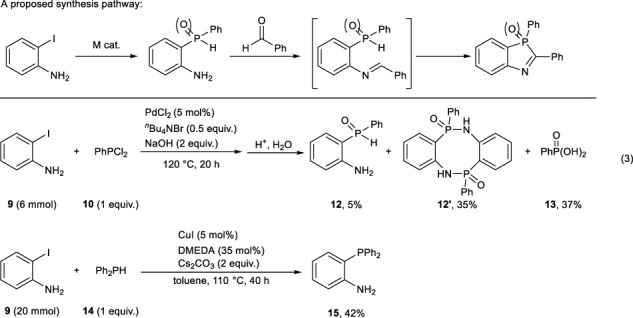

(3)



Then, we next examined the imine synthesis by dehydrative condensation from 2-aminophenyl-(diphenyl)phosphine **15** and benzaldehyde **16** (Table 1). When the condensation reaction of **15** and **16a** was conducted in the presence of molecular sieves at 25°C, the corresponding imine **17** was obtained in 12% yield (entry 1). Excess amounts of **16a** improved the yield of **17** (37%) (entry 3). Exchanging the solvent from toluene to methanol dramatically improved the yield (entry 5), and especially, 87% of the imine **17** was obtained when the reaction was conducted at 60°C (entry 6). However, attempted cyclization of the thus formed imine **17** using Pd(OAc)_2_ as a catalyst failed, and **17** was recovered as a phosphine sulfide **17'** (Eq 4).

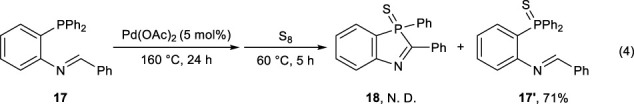

(4)



**TABLE 1:** Dehydrative condensation of 2-aminophenyl(diphenyl)phosphine **15** and benzaldehyde **16a**.

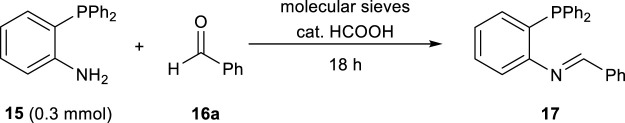				
Entry	Temp. (°C)	**16a** (equiv.)	Solvent	Yield **17** (%)^a^
1	25	1	Toluene	12
2	25	2	Toluene	15
3	25	4	Toluene	37
4	25	1	CH_3_CN	37
5	25	1	MeOH	64
6	60	1	MeOH	87

^a^ The yields were determined by ^31^P NMR spectroscopy.

Thus, we attempted the Pd(OAc)_2_-catalyzed cyclization using *in situ-*generated imine intermediate **17** (Eq. 5). The imine generation was performed using two methods. After treatment with sulfur, the corresponding 1,3-benzophosphole sulfide **18** was obtained in ca. 10% yields. However, most of the imine intermediate did not undergo the desired cyclization.

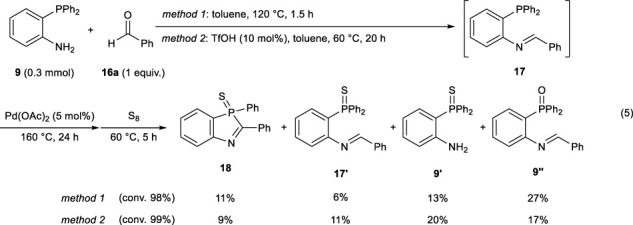

(5)



To promote cyclization by bringing palladium closer to the reaction site, we attempted to synthesize (2-bromophenylamino)methylphosphine intermediate using the three methods, as shown in Eqs. 6–8. The three methods involve *in situ* formation of imine intermediate, followed by the addition of P–H species to the formed N=C bond, and the desired (2-bromophenylamino)methylphosphine intermediates were obtained in good yields. After the –P(S)Ph_2_ group was reduced to the –PPh_2_ group, the cyclization was examined under the conditions of Pd(OAc)_2_ (10 mol%), DMF, 130°C, 12 h. Unfortunately, the desired cyclization did not occur.

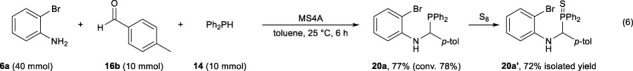

(6)



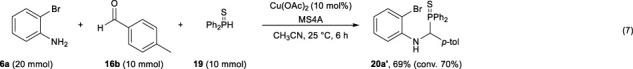

(7)



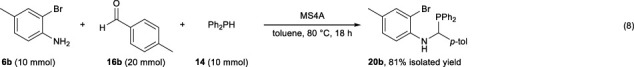

(8)



Since the Pd(OAc)_2_-catalyzed dephenylative cyclization was difficult, we next examined the cyclization using a nitrogen radical as a key species (Eq. 9). Diphenylphosphine **14** was added to trichloroacetonitrile **21** to form an imine derivative, which was oxidized with PIDA (PhI(OAc)_2_) to yield an imino radical. Radical cyclization and nucleophilic substitution of one chloro group by the acetoxy group afforded 1,3-azaphosphole derivative **22** in 27% yield.

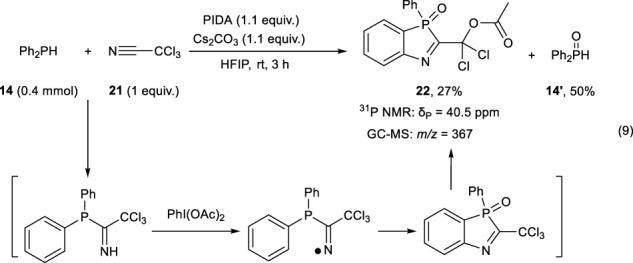

(9)



Since the aforementioned method did not achieve the construction of the 1,3-azaphosphole analogous skeleton, the known reaction was reviewed once, and the synthetic route was reconstructed (Berger et al., 2013). Phosphinic acid ester **24** is synthesized by C–P bond formation from 2-bromoaniline **6a** and PhP(O) (OEt)H **23** and then reduced with lithium aluminum hydride (LiAlH_4_) to synthesize disubstituted phosphines **25**. The synthesized phosphine **25** undergoes cyclization with acetylacetone or acetaldehyde, constructing the 1,3-azaphosphole analogous skeleton (Eq. 10).

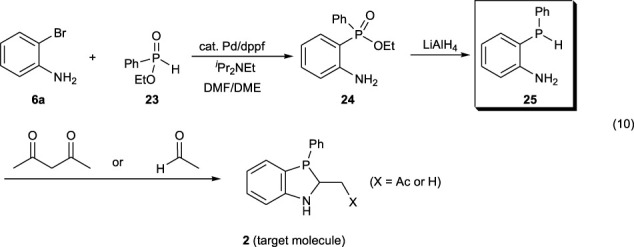

(10)



The palladium-catalyzed coupling reaction of 2-bromoaniline **6a** with PhP(O)(OEt)H **23** was examined. As shown in Eq. 11, the use of slightly excess amount of **6a** successfully resulted in good yield of the coupling product **24**.

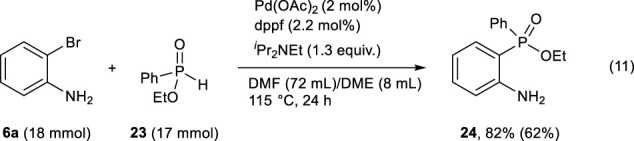

(11)



As we expected, the following reduction could be conducted using LiAlH_4_, and 2-(phenylphosphanyl)aniline **25** was obtained in 72% yield (Eq. 12).

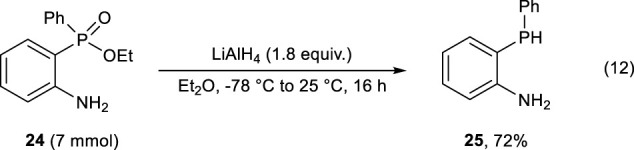

(12)



Surprisingly, 2-(phenylphosphanyl)aniline **25** is stable in air and can be purified by silica-gel column chromatography. Conventional synthetic methods of 1,3-benzoazaphosphole analogs require the use of reagents that are sensitive to air and moisture and highly toxic. In contrast, the use of air-stable **25** as a key synthetic intermediate provides a versatile and practical synthetic method for azaphospholes.

Phosphinic acid ester **24** could successfully be reduced using diisobutylaluminum hydride (DIBAL-H). As shown in Table 2, using excess DIBAL-H and prolonging the reaction time to 70 h dramatically improved the conversion of **24**, and **25** was obtained in up to 91% yield (entry 4).

**TABLE 2:** Optimization of the reaction conditions for the reduction of **24** using DIBAL-H.

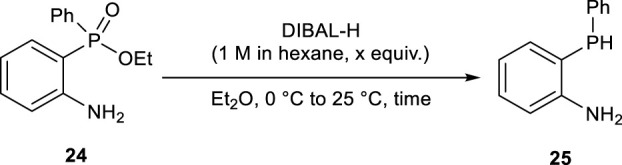				
Entry	**24** (mmol)	DIBAL-H (equiv.)	Time (h)	Yield of **25** (%)^a^
1	8	4.0	20	50
2	9	6.0	20	58
3	6	6.0	25	30
4	14	7.0	70	91
5	18	5.7	70	73

^a^ The yields were determined by ^31^P NMR spectroscopy.

The synthesized phosphine **25** was then allowed to react with acetylacetone in the presence of a catalytic amount of TsOH to synthesize **2**; however, the desired reaction did not proceed efficiently, and compound **26** was obtained in 30% yields along with the generation of many unidentified phosphorus-containing species (Eq. 13).

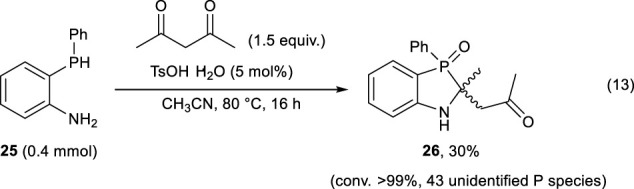

(13)



In contrast, when the solution of **25** (0.4 mmol) and acetaldehyde (1.2 equiv.) in toluene was heated at 120°C for 20 h, the desired 2-methyl-3-phenyl-2,3-dihydro-1*H*-benzo[*d*][1,3]azaphosphole **2** was successfully obtained in 75% yield (*anti*/*syn* = 43/57). Prolonging the reaction time improved the yield of **2**. Furthermore, a gram-scale synthesis of **2** was successfully achieved (Eq. 14).

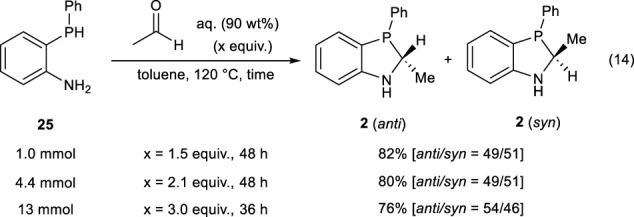

(14)



Moreover, we investigated the reactions of **25** with a variety of sulfurizing agents to synthesize 3-phenyl-2,3-dihydro-1*H*-benzo[*d*][1,3]azaphosphole-2-thione **3**, which is a synthetically interesting analogs of **2**. When carbon disulfide (CS_2_) was used as the sulfurizing agent, (2-aminophenyl)(phenyl)phosphanecarbodithioic acid **26** was formed in 48% yield along with some unidentified phosphorus byproducts (Eq. 15).

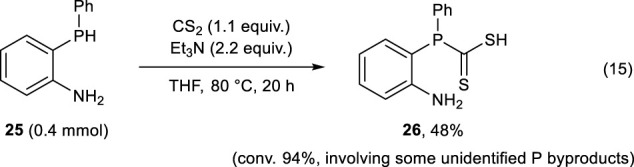

(15)



The reaction of **25** with 1,1′-thiocarbonyldiimidazole successfully afforded (**3)** in 50% yield, whereas the use of *O*,*O*-di- (pyridin-2-yl) carbonothioate resulted in the formation of (**3)** in 29% yield (Eqs. 16 and 17).

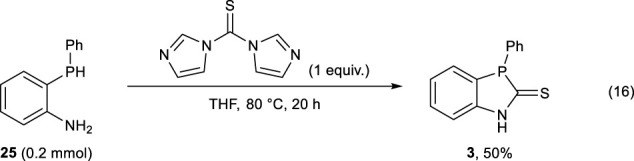

(16)



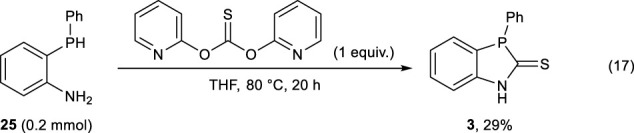

(17)



Encouraged by these results, we next investigated the scale-up synthesis of **3** using 1,1′-thiocarbonyldiimidazole. Although increasing the amount of 1,1′-thiocarbonyldiimidazole to 3 equiv. resulted in the formation of some unidentified compounds, using 1 equiv. of 1,1′-thiocarbonyldiimidazole and prolonging the reaction time to 48 h successfully afforded the desired **3** in up to 97% yield (Eq. 18).

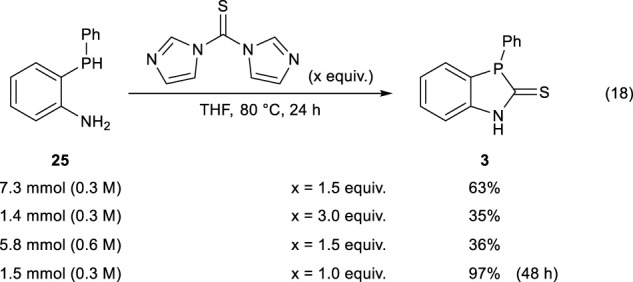

(18)



## 4 Conclusion

In this study, 1,3-benzoazaphosphole was selected as a target molecule, and various synthetic routes were investigated to establish a fundamental technology for the formation of five-membered heterocycles containing phosphorus and nitrogen through the introduction of a phosphorus functional group into the aromatic ring and the subsequent cyclization reaction. As a result, we found that 2-aminophenyl (phenyl)phosphine is an extremely promising synthetic intermediate with high stability and easy handling, and by using this air-stable phosphine as a key intermediate, we have succeeded in the practical synthesis of 3-benzoazaphosphole analogs, i.e., 2-methyl-3-phenyl-2,3-dihydro-1*H*-benzo[*d*][1,3]azaphosphole **2** and 3-phenyl-2,3-dihydro-1*H*-benzo[*d*][1,3]azaphosphole-2-thione **3**, for the first time.

We strongly hope that the synthetic strategy for azaphosphole derivatives presented in this study will be a new milestone in the construction of phosphorus-containing functional heterocycles and will significantly contribute to the further utilization of phosphorus-centered heterocyclic compounds in the field of organic synthesis and materials science.

## Data Availability

The original contributions presented in the study are included in the article/Supplementary Material; further inquiries can be directed to the corresponding author.
