# Supplementary material for: Practical synthesis of 1,3-benzoazaphosphole analogues
Source: Front Chem. 2023 May 25;11:1174895. doi: 10.3389/fchem.2023.1174895 (PMC10247990; doi:10.3389/fchem.2023.1174895)
Supplement: Supplementary file 1 [file DataSheet1.PDF]

# Supporting Information

# Practical Synthesis of 1,3-Benzoazaphosphol Analogues

Yuki Yamamoto[1], Soichiro Mita[1], Yuki Sato[1], Kentaro Yano[2], and Akiya Ogawa[1,*]

[1]*Department of Applied Chemistry, Graduate School of Engineering, Osaka Prefecture University, 1-1 Gakuen-cho, Nakaku, Sakai, Osaka 599-8531, Japan*

[2]*Functional Dye Division, Hayashibara Co., Ltd., 564-176 Fujita, Minami-ku, Okayama 701-0221, Japan*

ogawa@omu.ac.jp

## CONTENTS

| | page |
|---|---|
| Copies of NMR spectra of compound **24**, **25, 2**, and **3** | S2–S11 |

**Figure S1.** Copies of $^{1}H$ NMR, $^{13}C\{^{1}H\}$ NMR, and $^{31}P$ NMR spectra of **24**

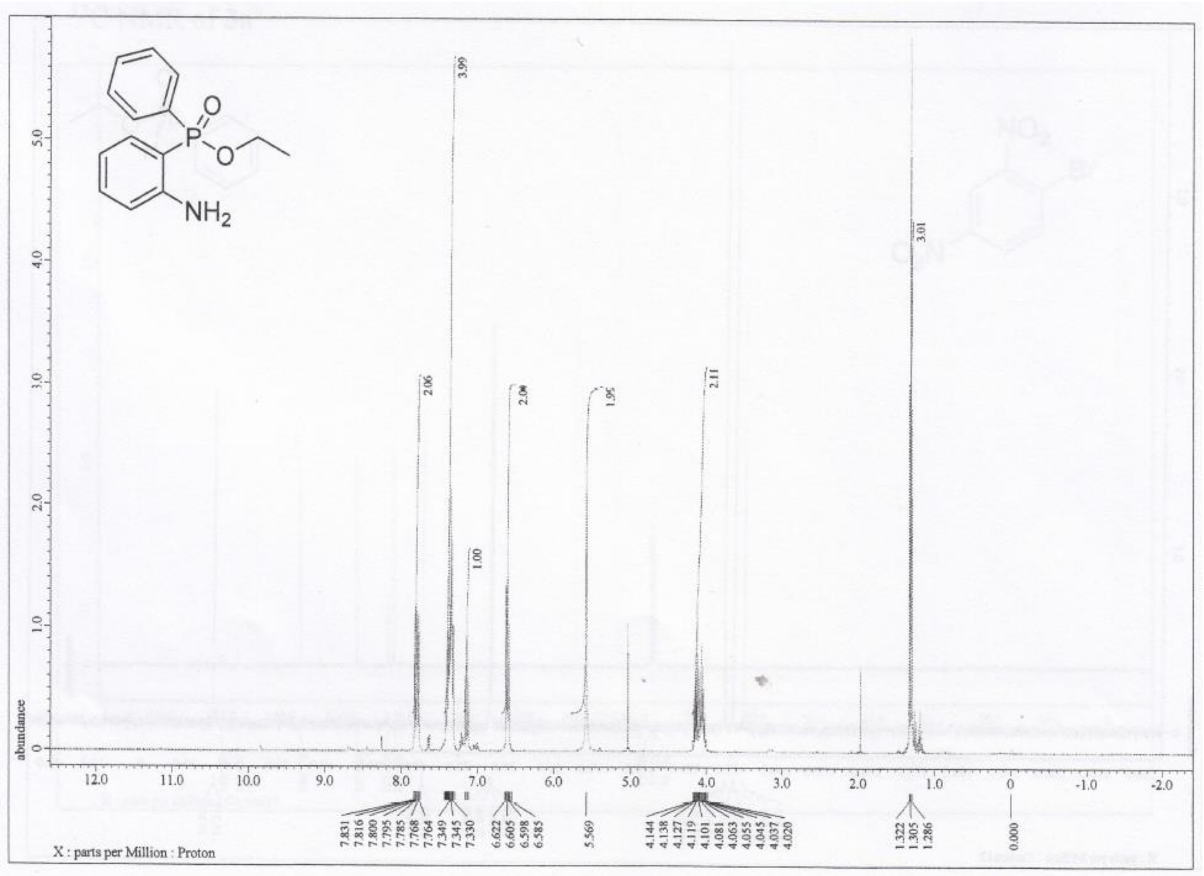

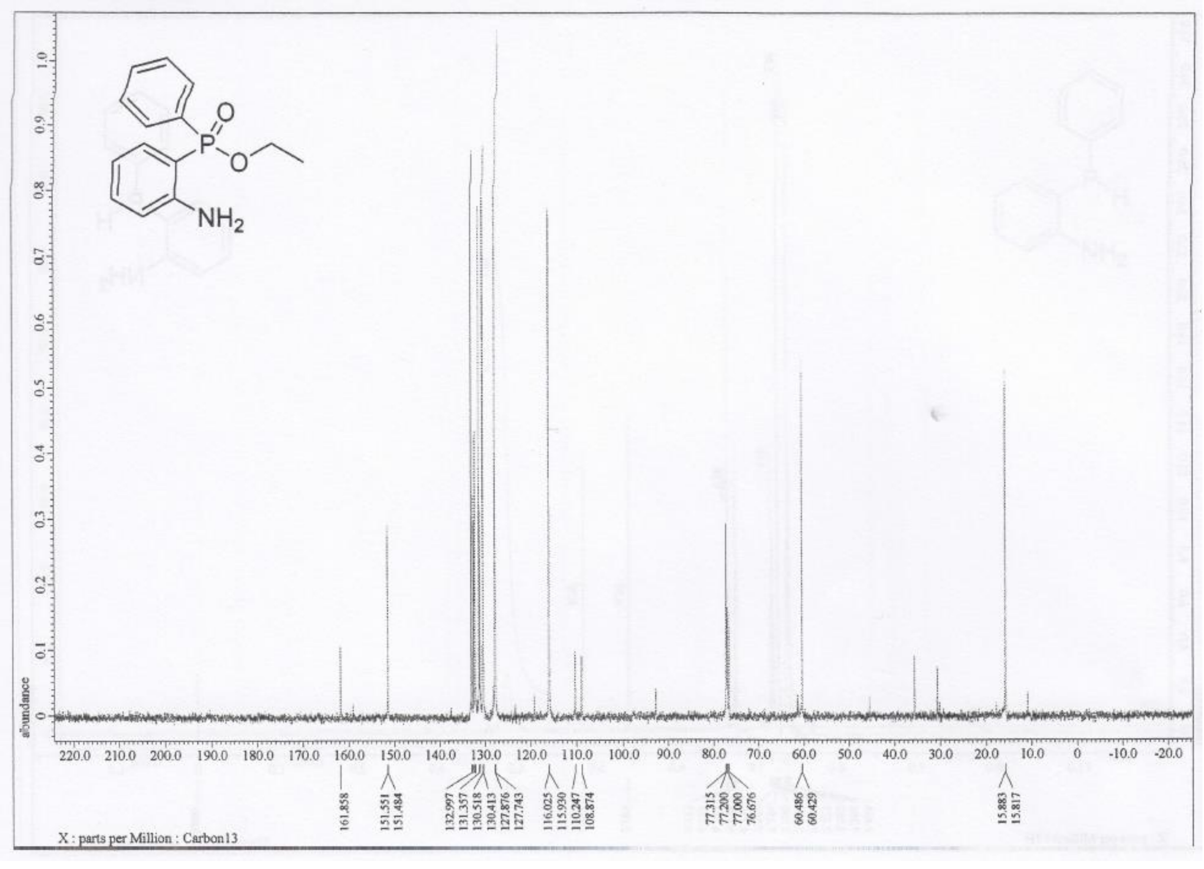

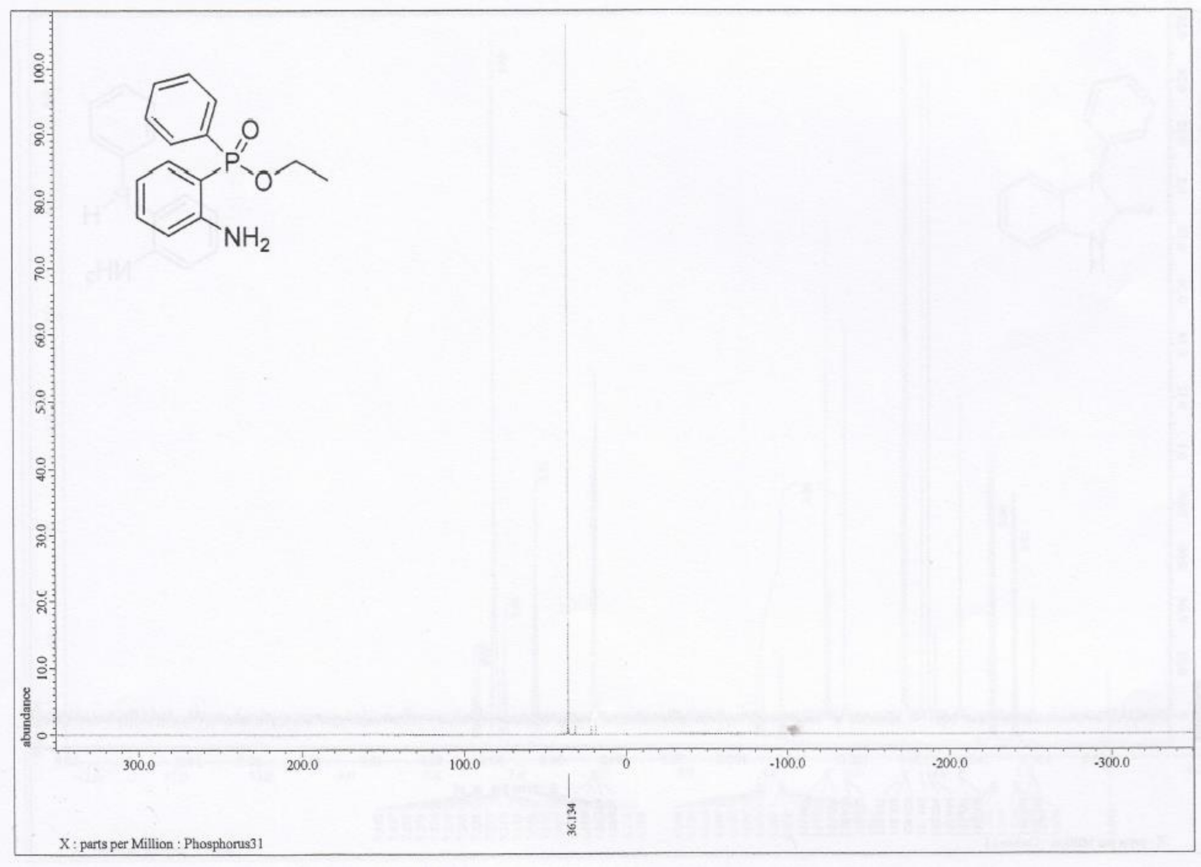

abundance
NH2
300.0
200.0
100.0
0
-100.0
-200.0
-300.0
36.134
X : parts per Million : Phosphorus31

**Figure S2.** Copies of $^{1}$H NMR, $^{13}$C{$^{1}$H} NMR, and $^{31}$P NMR spectra of **25**

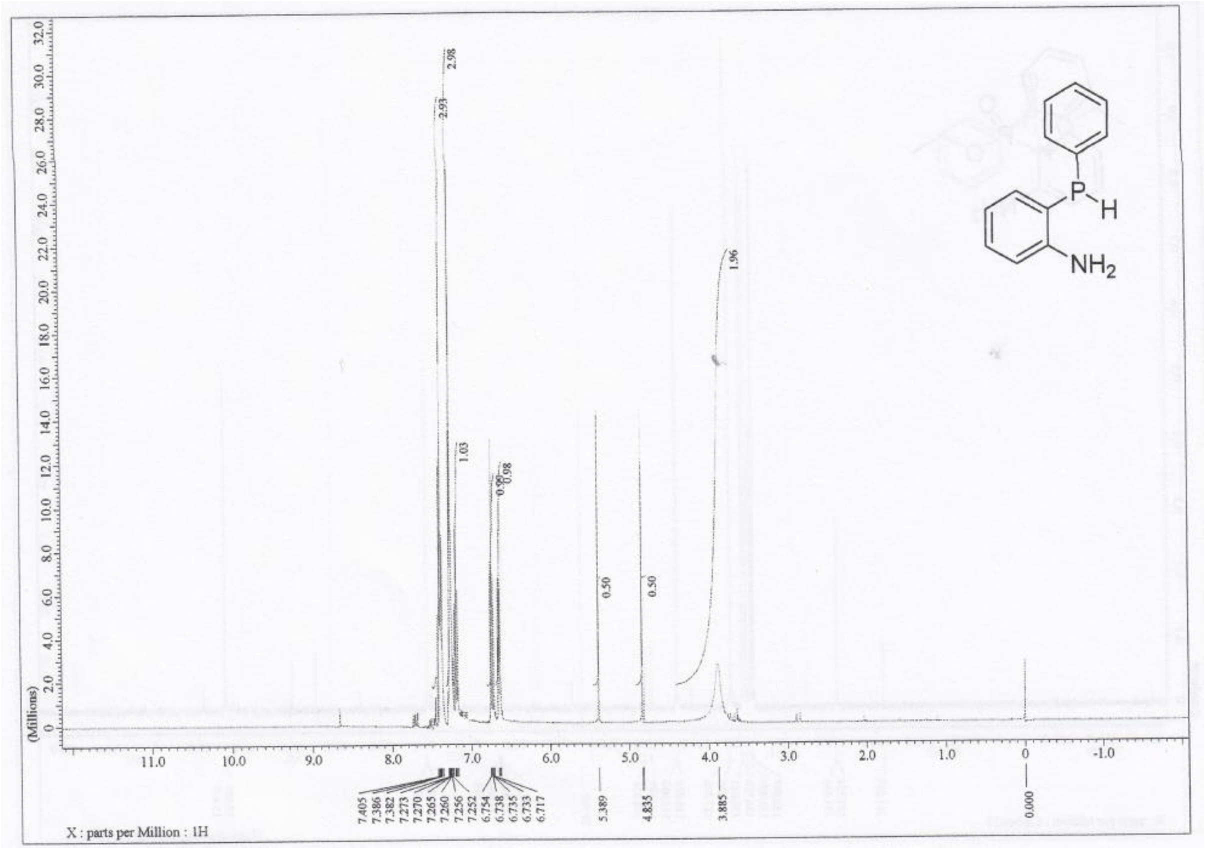

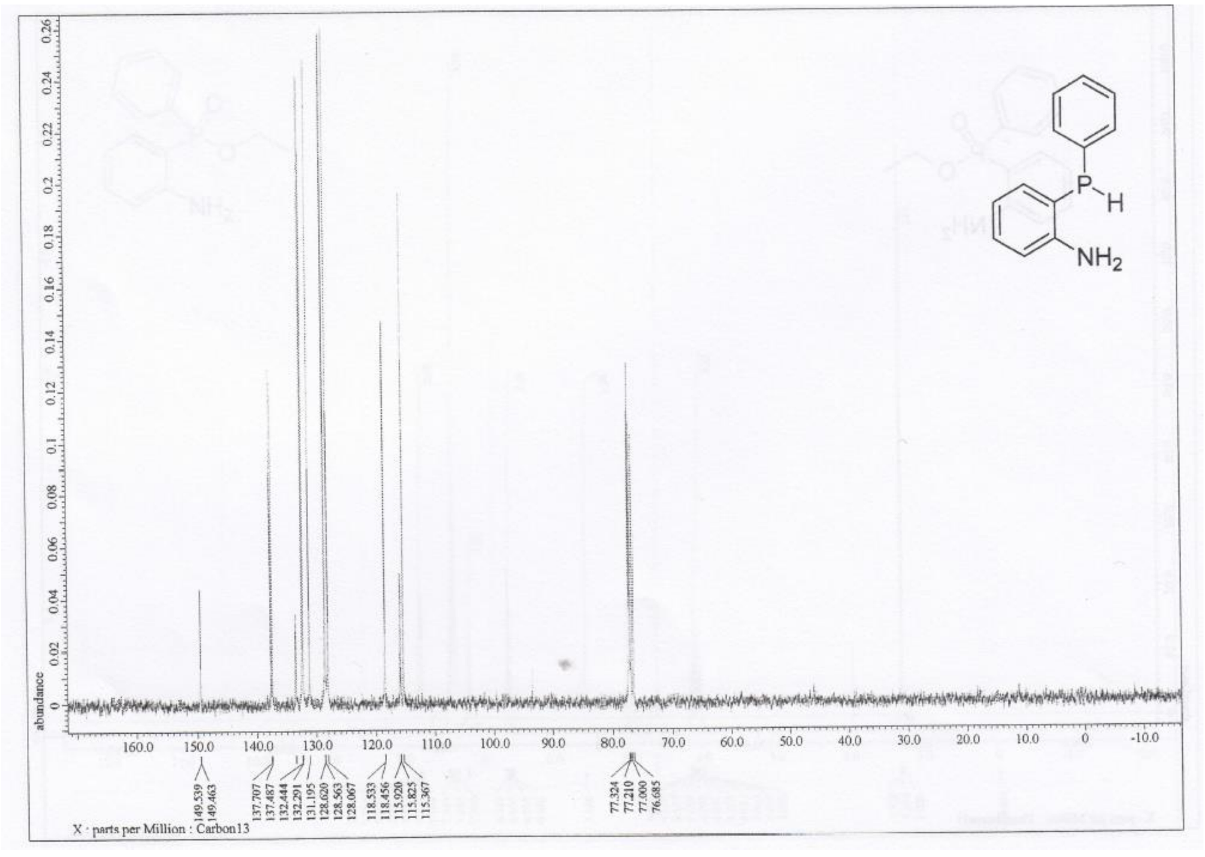

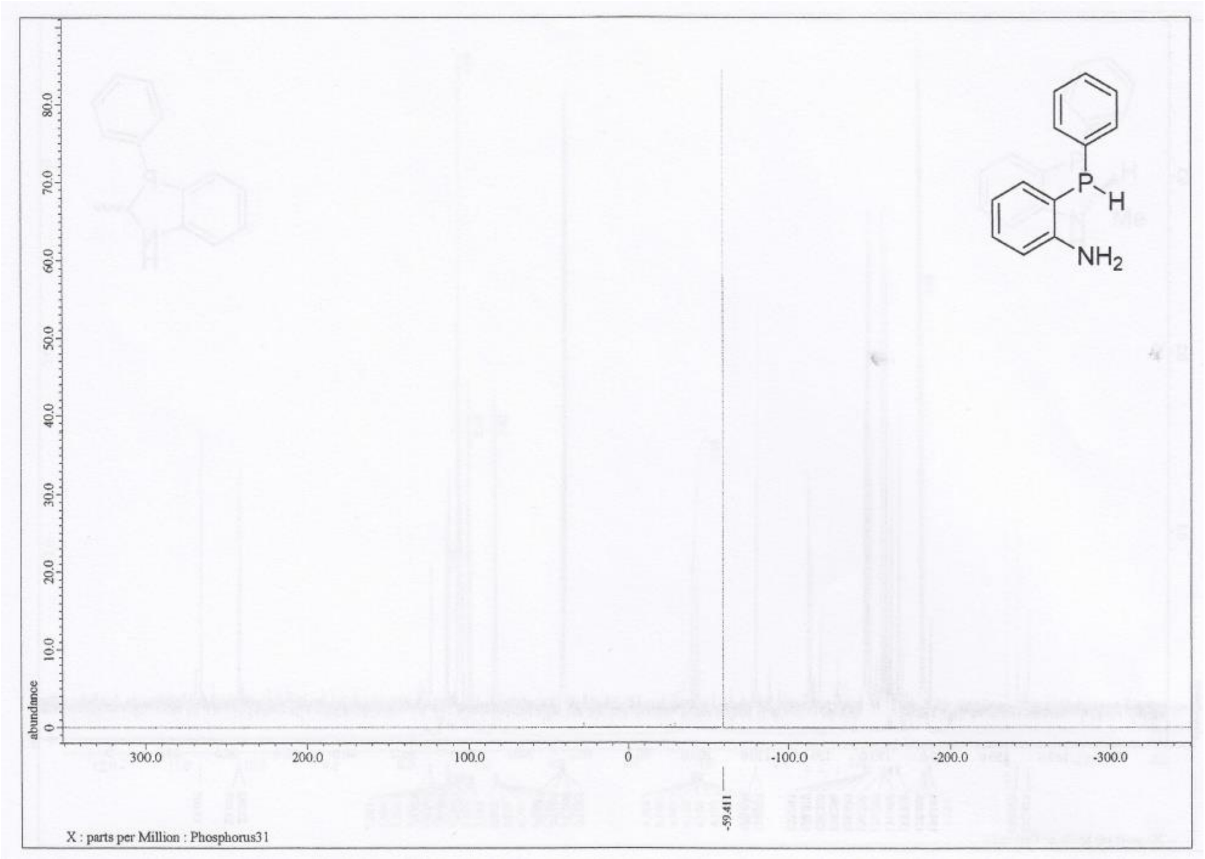

NH2
X : parts per Million : Phosphorus31
abundance
-59.411

**Figure S3.** Copies of $^{1}H$ NMR, $^{13}C\{^{1}H\}$ NMR, and $^{31}P$ NMR spectra of **2** (*anti*)

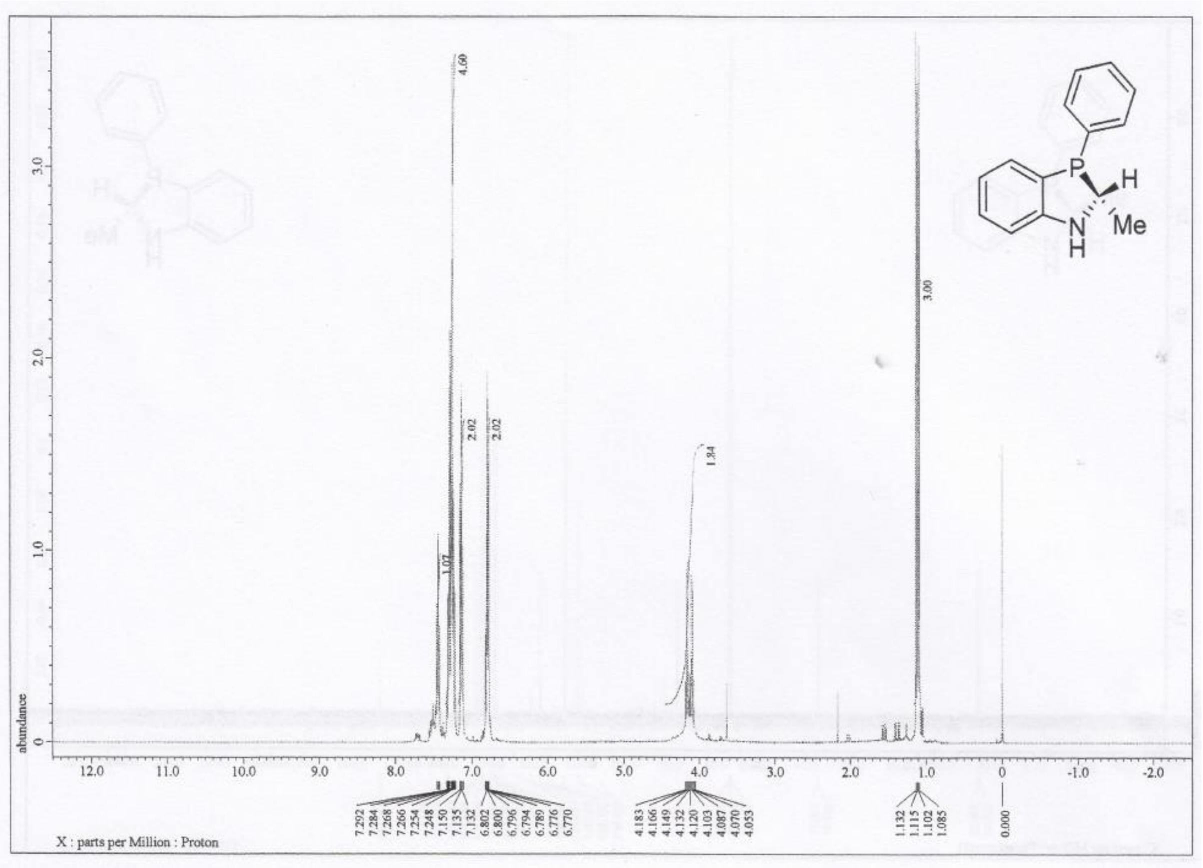

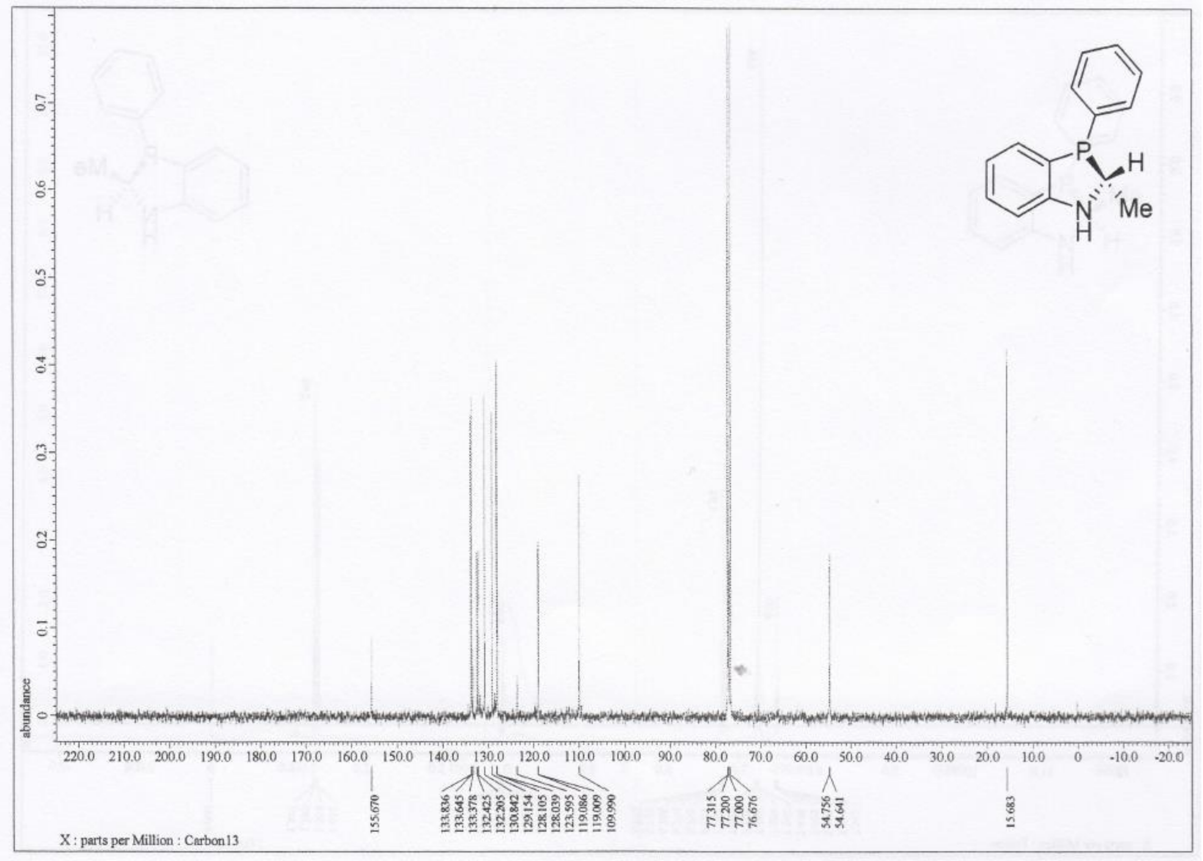

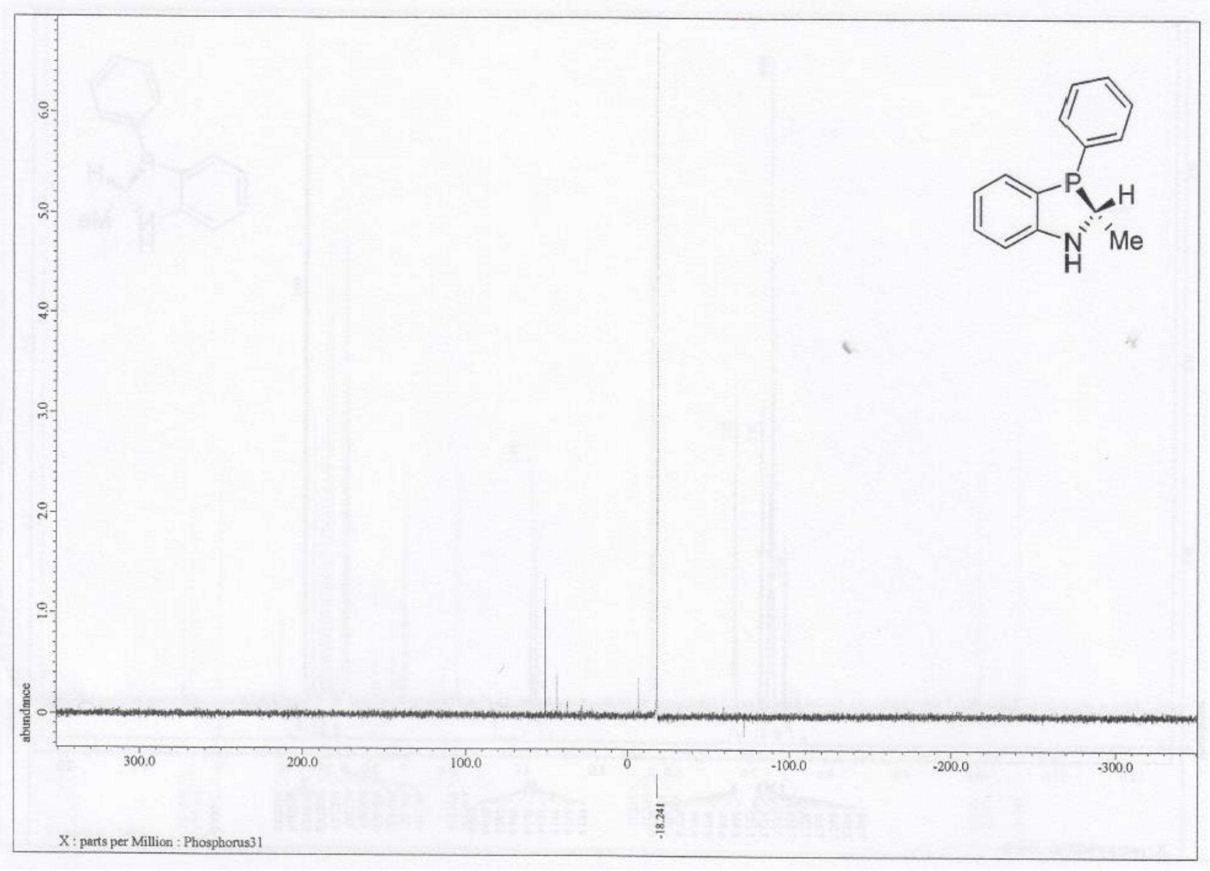

P
H
N
H
Me
abundance
0
1.0
2.0
3.0
4.0
5.0
6.0
300.0
200.0
100.0
0
-100.0
-200.0
-300.0
-18.241
X : parts per Million : Phosphorus31

**Figure S3.** Copies of $^{1}H$ NMR, $^{13}C\{^{1}H\}$ NMR, and $^{31}P$ NMR spectra of **2** (*syn*)

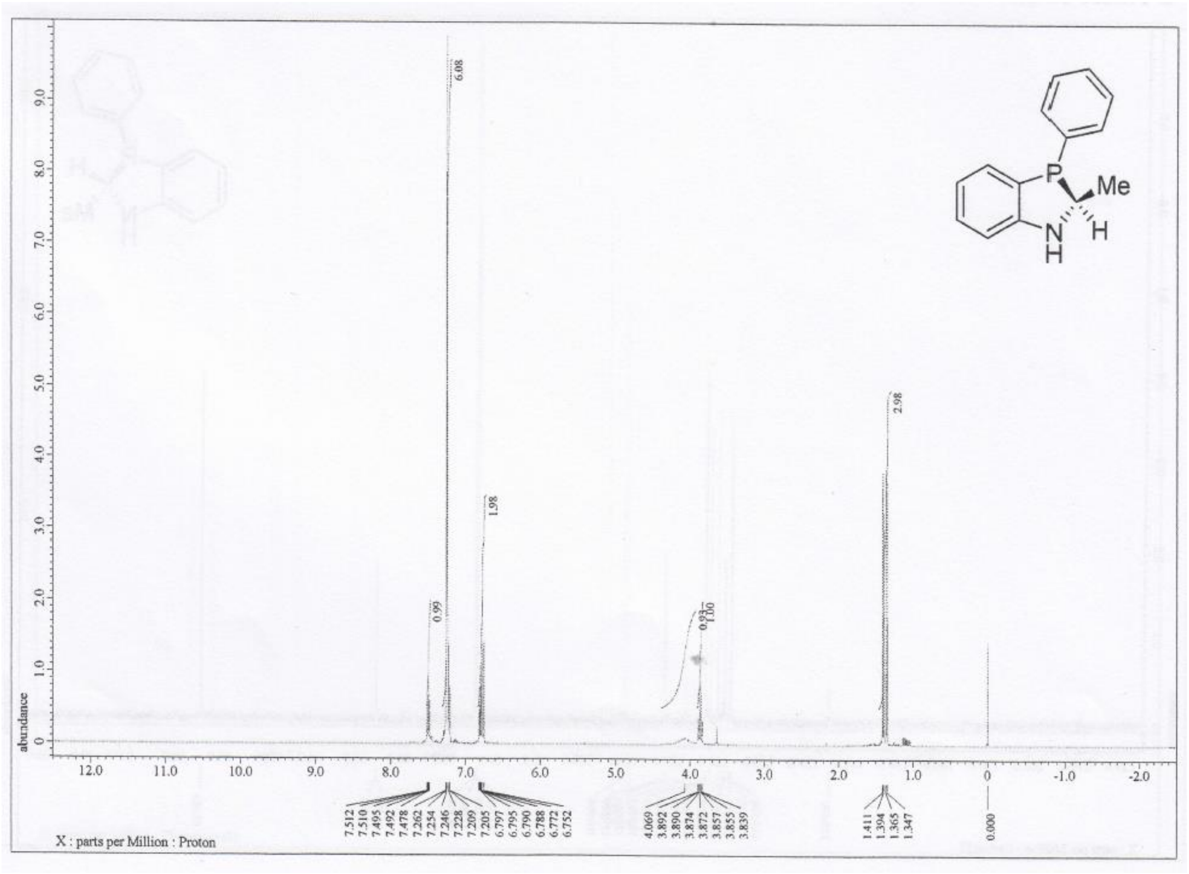


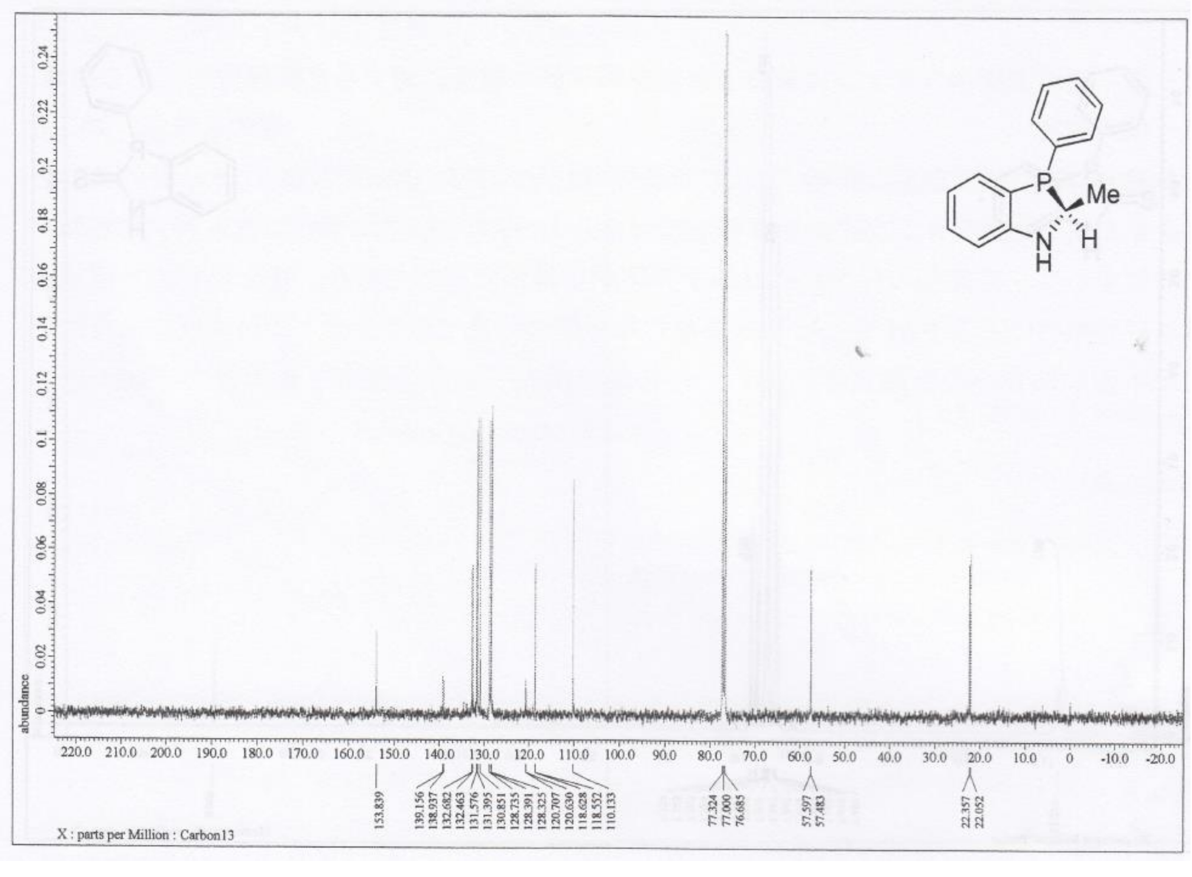

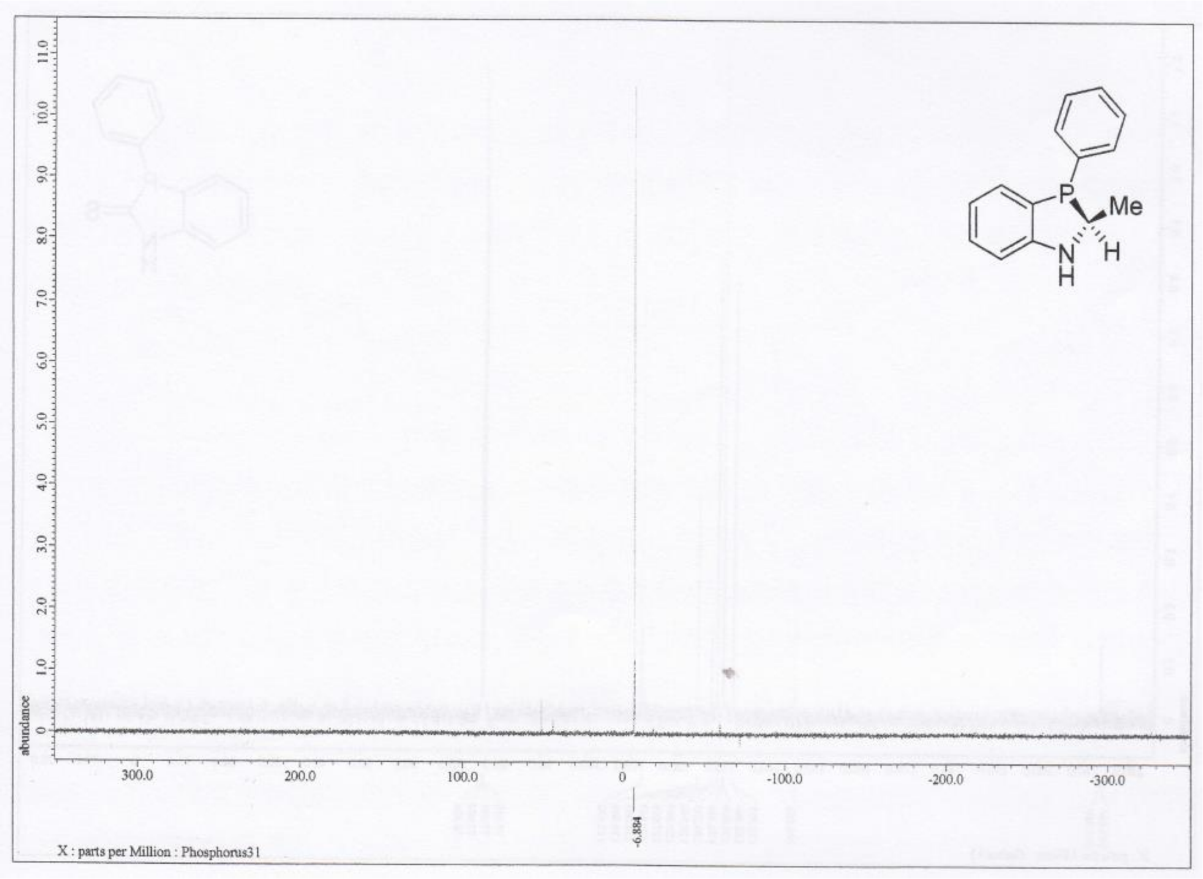
P
Me
N
H
H
abundance
X : parts per Million : Phosphorus31
-6.884

**Figure S3.** Copies of $^1H$ NMR, $^{13}C\{^1H\}$ NMR, and $^{31}P$ NMR spectra of **3**

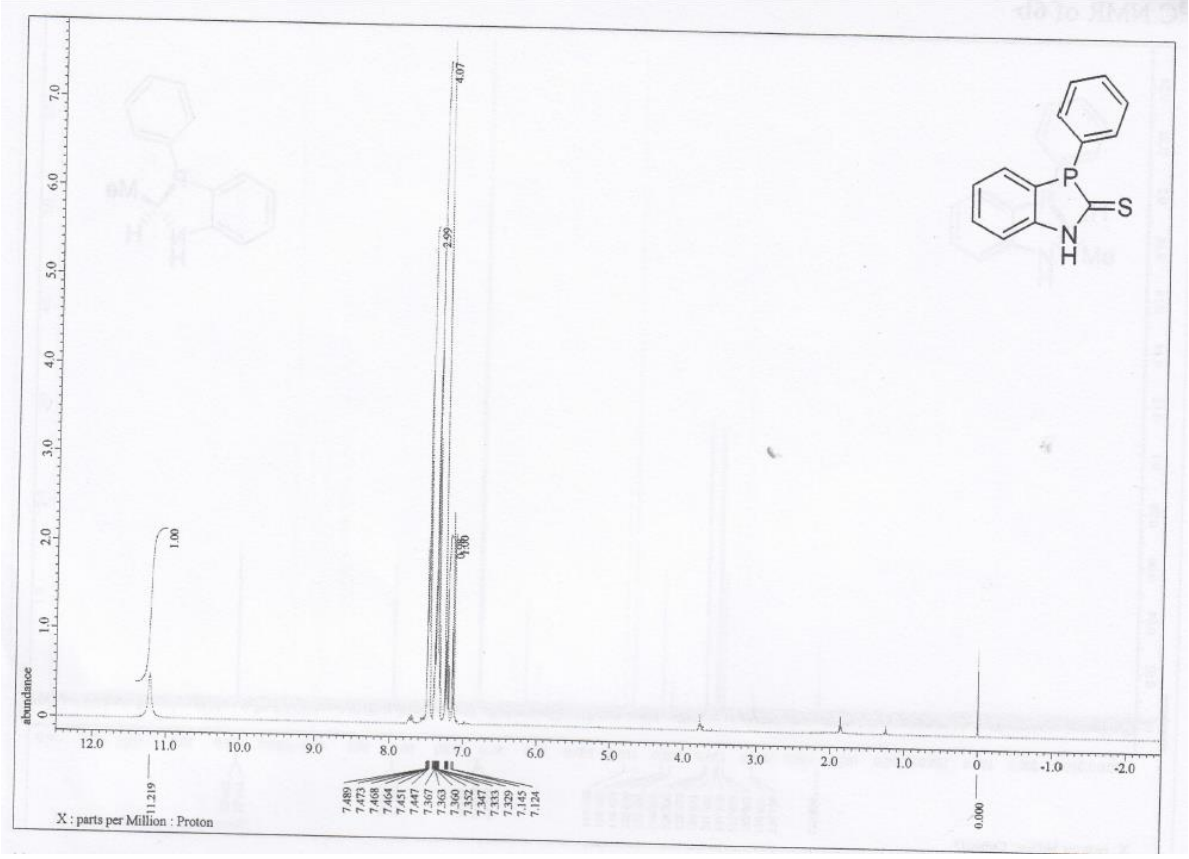

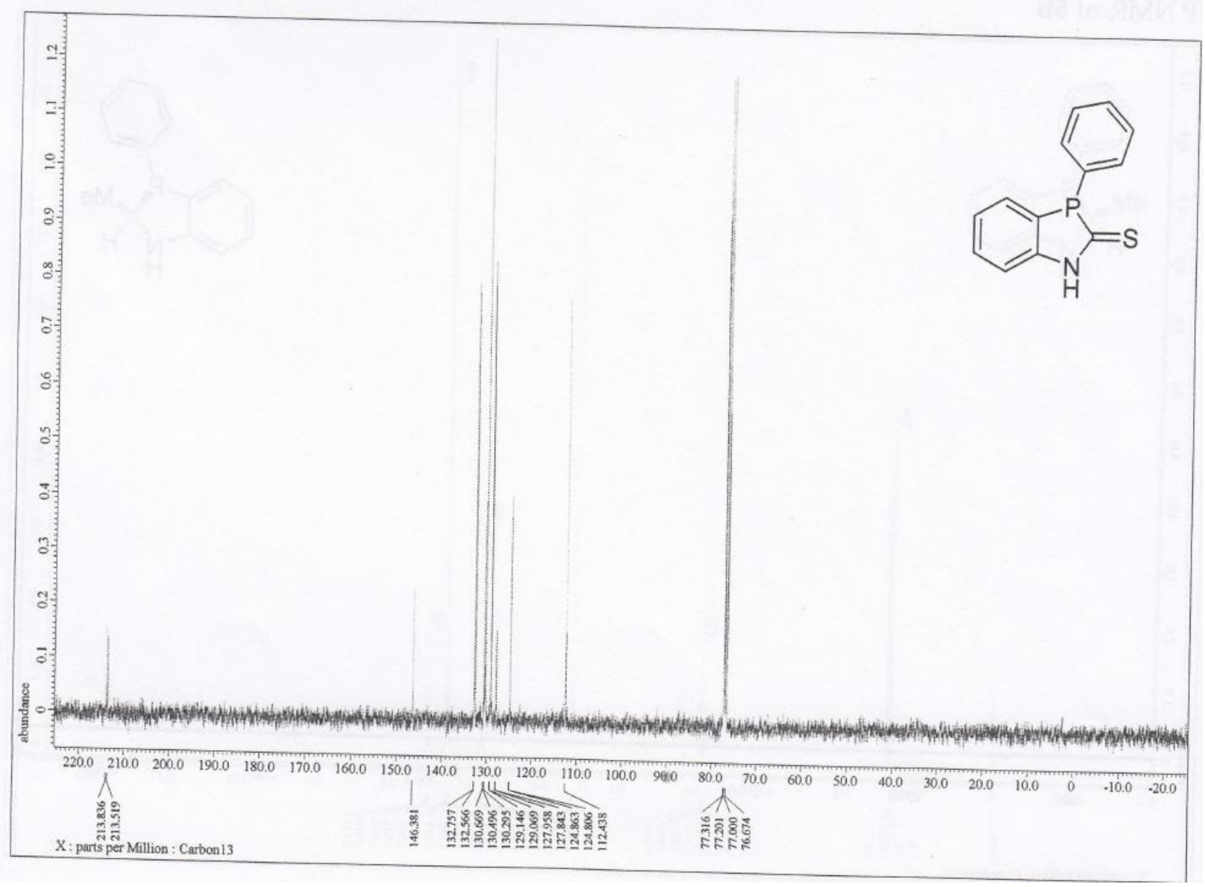

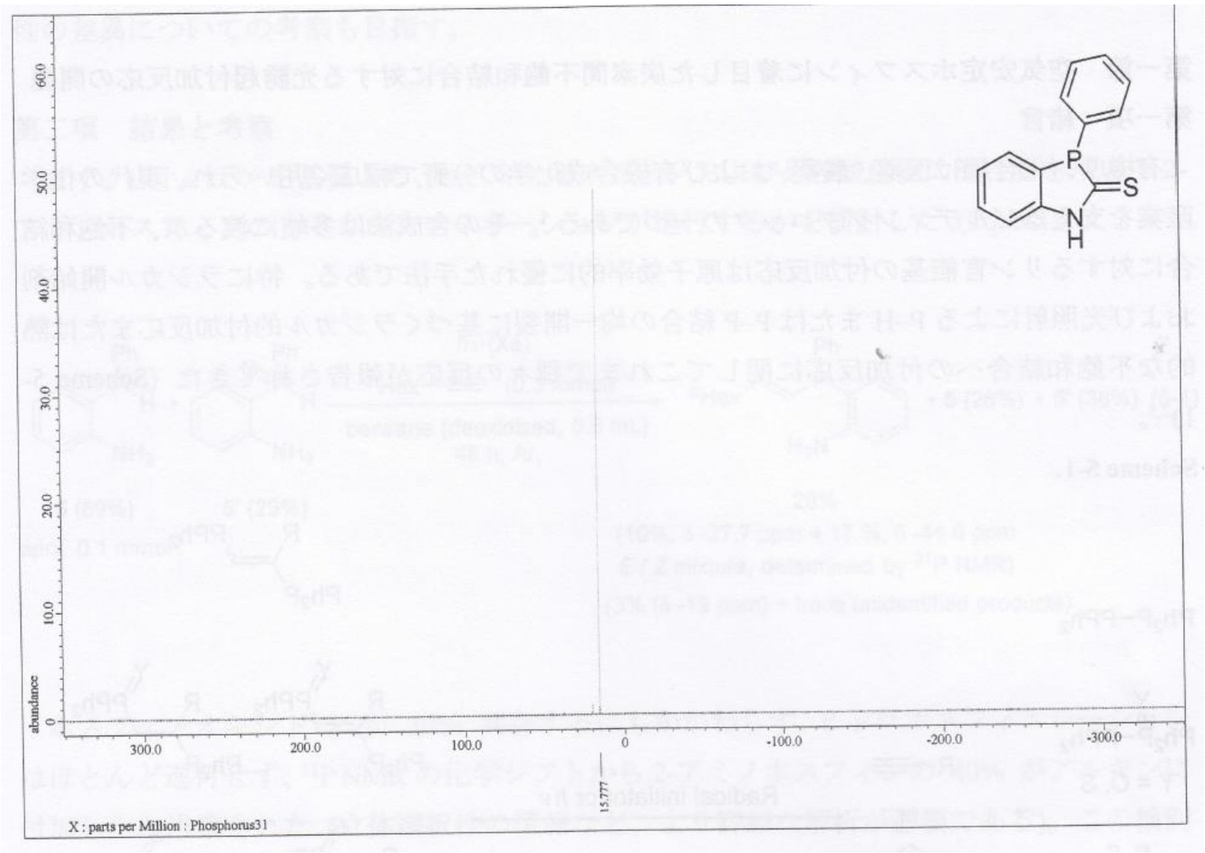

X : parts per Million : Phosphorus31
abundance
15.777
